# Addressing Mass Shootings in a New Light

**DOI:** 10.7759/cureus.9298

**Published:** 2020-07-20

**Authors:** Manal Ahmad, Hema Mekala, Judith Lone, Karlyle Robinson, Kaushal Shah

**Affiliations:** 1 Family Medicine, University of Manitoba, Winnipeg, CAN; 2 Psychiatry, Griffin Memorial Hospital, Norman, USA; 3 Psychiatry, Medical University of the Americas, Camps, KNA; 4 General Medicine, Medical University of the Americas, Camps, KNA

**Keywords:** mass shootings, guns, mental health, violence, mental illness, firearms

## Abstract

Locations change, casualties change, but the choice of weapon remains the same: firearms. With mass shootings gaining continuous limelight, we aim through this article to provide the readers an overview of the perpetrator's profile, along with various opinions proposed by the media, government, National Rifle Association (NRA), and health professionals. With mental health linked as a common factor for such incidents, we need to explore the different stances to eliminate such events. This article also provides a collaborative approach to alter the narrative and view it in a new light.

## Introduction and background

Mass shootings have shocked and mobilized the attitudes of the United States (US). It implies an incident involving four or more victims due to firearm-related injuries [1]. Despite not being a new phenomenon, they have been occurring throughout the nation since the 1900s [2]. It was also reported that the firearm homicide rate in the United States is 20 times higher than in other countries [3]. A report published in 2013 by Friedman and Michel's mentioned that in the United States, more than 31,000 deaths are related to gunshot wounds, of which 13,000 are due to homicide [3]. Whereas in 2017, the Center for Disease Control and Prevention (CDC) reported that about 39,773 people died from firearm-related injuries, of which 14,542 accounted for firearm-related homicides. It shows the increasing trends of mass shootings in the United States [4]. In the mass shootings that do occur, mental illness is often framed as the driving cause [5].

Literature searches were conducted by utilizing the MEDLINE database using keywords "mass shootings," "gun violence," in the context of "mental health," "National Rifle Association (NRA)," and "media". Abstracts and titles of the studies from January 1, 2008, to December 31, 2018, were meticulously reviewed that yielded 31 research articles. Our literature search included studies published in English and excluded studies published in any language other than English and those published before 2008. We identified eight relevant articles after reviewing the full text of all studies found through this indexed search and cross-referencing it with the study purpose. This review article focuses on how mental health is ascribed to be the root cause of horrific mass shootings and work that needs to be done to change that narrative collectively. The paper also aims to explore the risk factors for mass shootings and compares it to the various narratives proposed by the media, government, and healthcare.

## Review

Risk factors for mass shootings

There are many theories and explanations offered by the literature to understand the precipitating factors behind the mass shootings. According to the "Trinity of Violence" theory, the availability of firearms, a motivated murderer, and random targets are the risk factors behind violent massacres [6]. This theory suggests that while mental illness and anger are factors that precipitate a mass attack, it is more related to the perpetrator's personal experiences of tarnished self-worth that leads them to act aggressively [6]. Apart from mental illness, other factors that contribute to the violent mass shootings include substance abuse, anger issues, impulsive behavior, and discrimination in society [6]. Substance abuse is a crucial factor that could lead to violent behavior and aggression. Many offenders with a history of substance abuse and a criminal background often have minimal resources available to treat pre-existing mental illness [7]. In a report published in 2013 by Friedman and Michels stated that 4% of violence could be attributed to persons with mental illness [3]. In a paper published by Menninger in 2007, reported that, when an individual perceives a narcissistic injury and has no hope for achieving a reasonable resolution or cannot tolerate the injury further, then the individual feels a disregard for the consequence of violent acts [8]. Discrimination in the society leading to social rejection, along with conceived conviction of being bullied, which causes shooters to act out and avenge the injustice [9]. Even though it is rare, the contagion effect is where the perpetrator is seeking media's attention to gain fame by having their name broadcasted, and this could also potentially lead to mass shootings [6]. Lastly, the availability and easy access to weapons to shooters are ultimately critical factors in conducting mass violence [6,8].

Role of media

The world is connected through social media, including advertising, movies, print media, publishing, photography, news, radio, and television [10]. Mass shootings receive media's extensive and widespread attention, allowing public discourse to flow through. In reality, to what the media portrays, it is very complex to understand the mass murders. Three elements surrounding mass shootings that are usually highlighted by the media are firearm access and mental illness [6]. Sometimes, media uses high profile mass shootings where serious mental illness was a factor. For instance, a research study that analyzed 14 national and regional sources published from 1997 to 2012 mentioned that 17% of stories highlighted the cause of mass shootings as people with mental illness and 9% as gun violence. Whereas in the two weeks after mass shootings, these proportions increased to 33% and 25%, respectively [11]. Such coverage may influence the public population's sentiments, creating negative stereotypes, and stigmatism towards mental illness, leading them to generalize that all mass shootings are spurred from mental illness [3].

On the other hand, media encourages and supports gun restriction to those with a mental illness, as a quick and easy solution to mass shootings. Hence, there appears to be a disconnect between reforms in gun control and mental health as the stance media chooses to represent. This fact is supported and advocated by the US government and the NRA [9]. Despite the media's focus on gun control and safety, it does little to draw attention to gun reforms at the level of legislative law and policies [9].

Role of government and NRA

A mass shooting is an urgent call for gun control regulations. The United States has by far the highest number of gun ownership than any other nation in the world, with civilians owning 90 guns per 100 people, which accounts for about 270 million overall [12]. Research suggests that guns are the main determining factor in firearm-related deaths [3]. The US government has failed to enact appropriate gun reform legislation to counteract the easy access to firearms [13]. Since the 1990s, research related to gun-related violence has been discouraged [3]. They refrain from research linking guns to homicide, most likely for self-preservation [3]. During his term, former President Barack Obama issued an executive order for the Center for Disease Control and Prevention (CDC) to investigate the causes of gun violence prevention. As a result, the New York State passed a law, banned all assault weapons, and actively engaged in efforts to keep guns away from those who are mentally ill [3]. However, prohibiting all individuals with a history of psychiatric hospitalization from purchasing firearms with no adequate background checks was estimated to reduce the number of gun violence victims by 3% [12]. Some argue that gun control should be regulated to eliminate or reduce access, resulting in lower mass shootings; however, there is no research to support this, as the government has stopped the CDC to fund the project on gun violence [6]. Policies aimed at preventing rampage shootings remain controversial and not well-tested in the literature [6].

Mental health

The mental health system is in dire need of resource allocation for better treatment access [13]. When the media, government, and organizations like NRA provide a persuasive narrative that links the perpetrator involved in a rampage shooting with mental illness, this information creates the social stigma around mental health, leading to lower treatment rates and undesired adverse outcomes [7]. This narrative deviates the public perception of the cause of mass shootings from illegal firearm access to mental illness. It is crucial to change the attitudes the public has towards mental disorders since they have a distorted perception of the link between mental illness and mass shootings [7]. Although the rate of mental illness is increased amongst those participating is mass shootings, it is essential to note that not all mentally ill individuals are considered violent [6]. Mental illness can be regarded as one of the contributing factors, but it is inaccurate to make it the fundamental cause of shooting massacres [3].

While funding in mental health will allocate more resources to the experts for better screening and treatment of mental illness, it is critical to note the limited role of the behavioral health system plays in the overall scheme of healthcare. This system can prevent potential violence only in patients who have sought assistance voluntarily or willing to change and work on their mental illness [7].

## Conclusions

Mass shootings can happen anytime and anywhere in any given setting. A multifaceted approach that includes educating the public, regulating firearms, reducing barriers to seeking mental health services, and researching gun violence to produce effective policies might be beneficial. If such a mass massacre occurs in the future, we need to have a plan of action to address such a situation with an emergency team that consists of experts from around the nation to assist the impacted community and prevent future unfortunate events.
